# Dating Violence, Lifestyle and Risk of Type 2 Diabetes in Mexican Women University Students

**DOI:** 10.2174/0115733998283227240117060452

**Published:** 2024-01-30

**Authors:** Geu Mendoza-Catalan, José Alfredo Pimentel-Jaimes, Erika Nallely Orendain-Jaime, Claudia Jennifer Domínguez-Chávez, José Luis Higuera-Sainz, Alma Angélica Villa-Rueda, Ulises Rieke-Campoy, Adriana Camargo-Bravo

**Affiliations:** 1 Faculty of Nursing, Autonomous University of Baja California, Mexicali, Baja California, Mexico

**Keywords:** Dating violence, type 2 diabetes mellitus, risk, women, young adults, lifestyle

## Abstract

**Background:**

Dating violence is a prevalent issue among Mexican women, as is the incidence and prevalence of Type 2 diabetes mellitus (T2DM). The effects of dating violence can negatively impact lifestyle and, consequently, increase the risk of T2DM.

**Objective:**

This study aimed to explore the influence of dating violence on lifestyle and the risk of T2DM in women university students from Mexico.

**Methods:**

The study employed a cross-sectional and correlational design. The study population consisted of women university students. The sample size included 255 participants. Women aged 18 to 39 with current dating relationships and residency in Mexicali, Baja California, Mexico, were included. Data collection was conducted from February to May 2023. Correlations and multiple linear regression models were conducted.

**Results:**

A total of 255 women participated, with an average age of 21.6 years (SD = 3.2), and 32.2% had a history of intrafamily violence during childhood. 58.8% of the participants exhibited some level of risk of T2DM, and 56.7% of the lifestyle was mostly categorized as poor/fair. Detachment was the most prevalent type of dating violence, followed by coercion. Dating violence was correlated with lifestyle (r = -.430) and the risk of T2DM (r = .321). In the multiple linear regression model, dating violence influenced the risk of T2DM.

**Conclusion:**

Women who reported higher levels of dating violence have a less healthy lifestyle and a greater risk of T2DM. It is important to consider dating violence to improve lifestyle and prevent T2DM in Mexican women university students.

## 1 INTRODUCTION

Type 2 diabetes mellitus (T2DM) is one of the major health issues worldwide, affecting 8.8% of the global population, with a higher prevalence among women [1]. In Mexico, the prevalence of T2DM is higher in women than in men, and it is also the second leading cause of mortality in women [2, 3]. In recent years, there has been an increase in the prevalence of T2DM among young populations (<39 years old) in Mexico [4], which rose from 1.6% to 2.1% from 2016 to 2021 [3].

Several risk factors have been associated with the development of diabetes, including age, urbanization, lifestyle, body mass index (BMI), family history of diabetes, previous diagnosis of hypertension, consumption of sweetened drinks, smoking, alcohol consumption, sleep patterns, and mental health [5]. Nationally, 76.8% of women are overweight/obese, 19.5% report physical inactivity, 20.5% have a previous diagnosis of hypertension, and 21% have lipid disorders [3]. These statistics reflect the disparities that women face in terms of T2DM risk. However, other less explored factors may contribute to the increased vulnerability of Mexican women to T2DM.

Mexican women are expected to adhere to a set of values, behaviors, and norms associated with traditional femininity. Being an exemplary woman involves being submissive and subordinate, maintaining silence, and preserving family or partner harmony [6, 7]. These ideals of women are accepted by family and society, where it is common for women to sacrifice themselves for the sake of maintaining harmony with their partner, leading to greater emotional and mental health problems within the relationship [8, 9]. Under these societal expectations, women often consider economic dependency on their partners and the experience of living with intimate partner violence becomes something normalized [10, 11], which could negatively affect their lifestyle and risk of T2DM.

Intimate partner violence against women is another significant public health problem in Mexico. At the national level, 70.1% of women have experienced some form of violence, with 39.9% having suffered violence from their partners, mainly women aged 15 to 24 during dating. The primary forms of violence experienced by women include emotional or psychological violence, followed by physical, economic, and sexual violence; most women did not report or seek help [12]. The experience of violence increased during and after the COVID-19 lockdown [13]. Among the health problems experienced by women who suffer from violence are low self-esteem, depressive symptoms, anxiety, stress, post-traumatic stress, alcohol abuse, weight gain, suicidal ideation, and an unhealthy lifestyle [12, 14-17]. The implications of dating violence in women are also risk factors for developing T2DM [18-20]. Therefore, the presence of dating violence could be a risk factor for T2DM in women university students.

In light of the above, dating violence is a prevalent issue among Mexican women, as is the incidence and prevalence of T2DM. The effects of dating violence can negatively impact lifestyle and, consequently, increase the risk of T2DM. However, the influence of dating violence has not been studied in relation to the risk of T2DM despite its high prevalence in both health issues in Mexico. Therefore, this study aims to explore the influence of dating violence on lifestyle and the risk of T2DM in women university students from Mexicali, Baja California, Mexico.

## 2 MATERIALS AND METHODS

### 2.1 Study Design and Population

The study design was cross-sectional and correlational. The target population consisted of women university nursing students. The sample size was 255 participants, calculated using GPower 3.1 software with a 95% confidence level, 90% power, and an effect size of 0.09. Inclusion criteria were women aged 18 to 39, currently in dating relationships, and residents of Mexicali, Baja California, Mexico. Pregnant women and those with a previous diagnosis of T2DM or type 1 diabetes were excluded.

### 2.2 Data Collection

Data was collected from February to May 2023 using an online survey administered through Google Forms. The use of the online survey was facilitated through the Facebook social network, specifically on the official page of the nursing school. The instructions included the criteria for inclusion and exclusion. Additionally, access to the survey was limited to students with institutional email, allowing only one response per user. Physical bulletins were strategically placed in common areas within the institution's buildings. The invitation (physical and online) included a QR code that directed participants to the survey. Before answering the survey, the participant would read and accept the informed consent to proceed with the survey (Tables **1**-**4**).

### 2.3 Questionnaires

Dating violence was assessed using the Dating Violence Questionnaire [21]. This questionnaire consists of 20 questions and assesses various forms of violence, including coercion, sexual, physical, detachment, and humiliation. Each question is answered on a Likert scale ranging from 0 = Never to 4 = Always, with a total score ranging from 0 to 80 points. Higher scores indicate greater levels of dating violence. This questionnaire had a Cronbach's alpha of 0.91.

The risk of T2DM was measured using the Finnish Diabetes Risk Score (FINDRISC) Instrument [22]. This instrument assesses the probability of developing T2DM over the next 10 years. It comprises eight risk factors: age, BMI, waist circumference, physical activity, consumption of vegetables and fruits, medication use for hypertension, history of elevated blood glucose, and family history of diabetes. The total score is interpreted as follows: <7 low risk, 7-11 slightly elevated risk, 12-14 moderate risk, 15-20 high risk, >20 very high risk. The sensitivity and specificity of the questionnaire for diagnosing T2DM are 87.50% and 52.55%, respectively.

Lifestyle was assessed using the fantastic questionnaire [23]. This questionnaire comprises 25 questions divided into 10 dimensions: family and friends, physical activity, nutrition, tobacco, alcohol, sleep/stress, personality, interior, work, and drugs. Response options range from 0 to 2. The sum of all responses is converted to a scale from 0 to 100 points, with higher scores indicating a better lifestyle. Scores are categorized as <60 points (poor lifestyle), 60 to 69 (fair), 70 to 84 (good), and >85 (excellent). The questionnaire had a Cronbach's alpha of 0.82.

### 2.4 Data Analysis

The data was downloaded in Excel from Google Forms and subsequently transferred and analyzed using SPSS v. 27 software. Descriptive analysis, including frequencies and percentages, was employed for categorical variables, while continuous variables were analyzed using measures of central tendency and dispersion (mean and standard deviation). In the bivariate analysis, Spearman's correlation coefficient was used. In the regression model for lifestyle, the introduction method was used with age, children, employed, socioeconomic status, intrafamily violence, partner age, partner occupation, relationship duration, sexual orientation, semester and dating violence as predictor variables. For the multiple linear regression model for diabetes risk, a hierarchical model was used, in which, in the first step, the variables age, children, employed, socioeconomic status, intrafamily violence, partner age, partner occupation, relationship duration, sexual orientation, semester and dating violence were included. In the second step, the lifestyle variable was included to identify the change in the effect of dating violence on the risk of diabetes.

## 3 RESULTS

### 3.1 Sociodemographic Characteristics

A total of 255 women participated in the study, with an average age of 21.6 years (SD = 3.2), and the average current semester of study was 4.1 (SD = 1.9). The majority perceive themselves as heterosexual, do not have children, have a middle socioeconomic status, and their parents live together. The average age of their partners was 23.4 years (SD = 4.9). Their partner's occupation was employed (full-time), and the average duration of the relationship was 28.6 months (SD = 31.2), as shown in Table **1**.

### 3.2 Description of Dating Violence, Lifestyle and Risk of T2DM

According to the description of the risk of T2DM, 58.8% of the participants exhibited some level of risk, mostly a slightly elevated risk. Concerning the type of dating violence, the highest scores were reported for detachment and coercion. Lastly, the lifestyle was mostly categorized as poor/fair, as shown in Table **2**.

### 3.3 Correlation Analysis

In the bivariate analysis, the total score of dating violence, as well as its sub-dimensions, positively correlated with the risk of T2DM and negatively with lifestyle, as presented in Table **3**.

### 3.4 Multiple Linear Regression Analysis

In the lifestyle model, sexual orientation (bisexual, lesbian and pansexual) and dating violence explained 18.5% of the variance (F = 20.166, *p* = 0.000). On the other hand, in the first model for T2DM risk, having children, current work, sexual orientation (bisexual, lesbian and pansexual), and dating violence explained 12.1% of the variance (F = 9.732, *p* = 0.000). When including the lifestyle variable, the explained variance of T2DM risk increased to 20% (F = 6.289, *p* = 0.000), as shown in Table **4**.

## 4 DISCUSSION

This study found that dating violence influences lifestyle and the risk of T2DM in women university students in Mexicali, Baja California, Mexico. More than half of the students showed some level of T2DM risk, which is higher than in previous studies conducted with Mexican populations [24, 25]. Nationally and internationally, women are a vulnerable group for the incidence, prevalence, and mortality associated with T2DM [1, 3]. Compared to men, women have more risk factors such as obesity, sedentary lifestyles, eating disorders, mental health issues, and hypercholesterolemia. Additionally, the risk increases for women during pregnancy due to weight gain and gestational diabetes [26].

Lifestyle was predominantly classified as poor to fair, consistent with previous studies [27, 28]. University students often face high academic demands and spend more time on school activities, leaving less time for leisure and leading to low self-care and unhealthy lifestyles, thereby increasing the risk of developing chronic diseases such as T2DM [29]. Furthermore, in the case of women, traditional family ideas persist today that women must fulfil various roles, such as performing domestic activities at home, from which men are excluded. Therefore, university women face greater pressure to fulfil their academic responsibilities and household duties, affecting their lifestyle [30].

According to dating violence, the main types of violence that emerge are detachment and coercion, similar to other research [31, 32]. Women university students tend to report a higher tolerance for coercive and detached behaviors from their partners [33]. For many years, women have been taught that they should tolerate their partner's behaviors and are held responsible for the success of a relationship [14, 34]. In such situations, men's behaviors during the relationship may be considered problematic but not necessarily violent [35, 36]. These behaviors are often overlooked because women perceive them as inconsistent, temporary, and nearly invisible, similar to microaggressions [36, 37]. Over time, violence, dominance, and manipulation gradually escalate, especially in women with emotional dependence [38, 39].

Our results show that women who reported higher levels of dating violence exhibited a less healthy lifestyle, which is consistent with other studies [17, 39]. In dating violence relationships, they have been characterized as power dynamics, where the perpetrator creates a system of dominance and control over the partner's decisions and actions [40]. In this context, the perpetrator can induce isolation of the partner from their social life and daily activities. Within these violent behaviors, the perpetrator exerts control over the partner's clothing choices, restricts activities, and may even enforce restrictions on communication with friends or family [31, 41]. During dating violence, women have difficulties seeking help due to the fear that the violent behaviors from their partners may escalate [17].

Finally, dating violence influenced the increased risk of T2DM in Mexican university women; however, the impact of dating violence decreased when lifestyle factors were taken into account. Thus, dating violence should be considered an independent variable in the risk of T2DM [42-44], and some authors have explained the linkage between partner violence and T2DM through psychological consequences and biochemical alterations. For example, due to the mental health issues caused by dating violence, elevated levels of cortisol and C-reactive protein have been identified, which could lead to a constant and prolonged inflammatory process, ultimately resulting in insulin production deficiency [45-47]. Additionally, other studies have reported that women experiencing higher levels of violence are more likely to have hyperglycemia [48]. On the other hand, women experiencing partner violence can also develop eating disorders due to low self-esteem, anxiety, and depression, which may lead to increased consumption of high-fat, high-carbohydrate, and sugary foods [47, 49], resulting in weight gain [15, 50]. Hence, it should be noted that exposure to violence in women may increase the risk of T2DM through psychological, behavioral, and biochemical implications. Programs aimed at reducing and preventing violence against women need to consider clinical assessments of diabetes risk and the inclusion of content to improve lifestyle [51].

This study has some limitations. As a weakness, it is a cross-sectional study, so causation cannot be determined between the study variables. The results of this study can only be generalized to the study population. As a strength, it is one of the first empirical (primary) studies to analyze the variables of dating violence and the risk of T2DM in Mexican women university students.

## CONCLUSION

Mexican women university students who report higher levels of dating violence show a less healthy lifestyle and a higher risk of T2DM. It is crucial to consider the prevention and reduction of partner violence in women to promote healthier lifestyles and reduce the risk of developing T2DM in the future.

## AUTHORS’ CONTRIBUTIONS

The authors confirm contribution to the paper as follows: Study Concept or Design: Geu Mendoza Catalán; Data Collection: José Alfredo Pimentel-Jaimes, Erika Nallely Orendain-Jaime, Claudia Jennifer Domínguez-Chávez, Adriana Camargo-Bravo & Alma Angélica Villa-Rueda; Data Analysis or Interpretation: José Luis Higuera-Sainz & Ulises Rieke-Campoy. All authors reviewed the results and approved the final version of the manuscript.

## Figures and Tables

**Table 1:** Characteristics of the participants and their partners.

**-**	**ƒ**	***%***
Age	21.6 (3.2)	
Semester	4.1 (1.9)	
Children		
Yes	29	11.4
No	226	88.6
**Employed (part time)**	**-**	**-**
Yes	88	34.5
No	167	65.5
**Socioeconomic Status**	**-**	**-**
Low	38	14.9
Middle	217	85.1
High	-	-
**Intrafamily Violence**	**-**	**-**
Yes	82	32.2
No	173	67.8
**Marital Status of Parents**	**-**	**-**
Live together	153	60.0
Divorced	37	14.5
Separated	47	18.4
Abandoned	18	7.1
Sexual Orientation		
Heterosexual	215	84.3
Bisexual	29	11.4
Lesbian	4	1.6
Pansexual	7	2.7
**Partner**	-	-
Age *M(SD)*	23.4 (4.9)	
**Occupation**	**-**	**-**
Student	53	20.8
Employed (Full time)	105	41.2
Student and employed (part time)	97	38.0

**Note:** M = Mean, SD = Standard Deviation, f = Frequency.

**Table 2:** Description of dating violence, lifestyle, and type 2 diabetes mellitus risk.

**-**	**ƒ **	***%***
T2DM Risk M(SD)	8.6(4.7)	-
Low	105	41.2
Slightly elevated	97	38.0
Moderate	21	8.2
High	28	11.0
Very high	4	1.6
Dating Violence M(SD)	5.1 (8.0)	-
Coercion M(SD)	1.3 (2.5)	40.4%
Sexual M(SD)	0.5 (1.5)	21.6%
Physical M(SD)	0.2 (0.9)	12.2%
Detachment M(SD)	2.2 (3.3)	58.8%
Humiliation M(SD)	0.8 (1.9)	29.0%
Lifestyle M(SD)	66.8 (11.6)	-
Poor	55	21.6
Fair	87	34.1
Good	103	40.4
Excellent	10	3.9

**Note:** M = Mean, SD = Standard Deviation, f = Frequency, T2DM = Type 2 Diabetes Mellitus.

**Table 3:** Correlation analysis between dating violence, lifestyle, and T2DM risk.

**-**	**T2DM Risk**	**Dating Violence**	**Coercion**	**Sexual**	**Physical**	**Detachment**	**Humiliation**
T2DM risk	-	0.321**	0.226**	0.121*	0.147*	0.295**	0.179**
Lifestyle	-0.344**	-0.430**	-0.295**	-0.341**	-0.186**	-0.398**	-0.330**

**Note:** *p<.05, **p<.01, T2DM = Type 2 Diabetes Mellitus.

**Table 4:** Multiple linear regression for lifestyle and T2DM risk.

**-**	**Model**	**Model T2DM Risk**	
	**Lifestyle**	**Step 1**	**Step 2**
Variable	β	β	β
Age	0.045	-	-
Children (yes)	-0.027	0.185*	0.153*
Employed (yes)	-0.087	0.024*	0.010
Socioeconomic Status	0.057	-0.027	-0.027
Intrafamily Violence	-0.087	0.034	0.043
Partner's Age	0.090	0.088	-0.007
Partner's Occupation	0.051	0.055	0.077
Relationship duration	-0.007	0.060	-0.101
Sexual orientation	-0.146*	0.183**	0.100
Semester	-0.051	-0.108	-0.095
Dating violence	-0.364**	0.240**	0.125*
Lifestyle	-	-	-0.350**

**Note:** *p<.05, **p<.01, T2DM = Type 2 Diabetes Mellitus.

## Data Availability

The data supporting this study's findings are available from the corresponding author, GMC, on special request.

## LIST OF ABBREVATIONS

T2DM: Type 2 Diabetes Mellitus
BMI: Body Mass Index
FINDRISC: Finnish Diabetes Risk Score

## STANDARDS OF REPORTING

STROBE guidelines were followed.
